# Successful post-glacial colonization of Europe by single lineage of freshwater amphipod from its Pannonian Plio-Pleistocene diversification hotspot

**DOI:** 10.1038/s41598-020-75568-7

**Published:** 2020-10-29

**Authors:** Hedvig Csapó, Paula Krzywoźniak, Michał Grabowski, Remi Wattier, Karolina Bącela-Spychalska, Tomasz Mamos, Mišel Jelić, Tomasz Rewicz

**Affiliations:** 1 Institute of Oceanology Polish Academy of Sciences, 81-712 Sopot, Poland; 2 Faculty of Biology and Environmental Protection, Department of Invertebrate Zoology and Hydrobiology, University of Lodz, 90-237 Lodz, Poland; 3 Faculty of Sciences, Department of Hydrobiology, University of Pecs, Pecs, 7634 Hungary; 4 UMR CNRS 6282 Biogéosciences, Université Bourgogne Franche Comté, 21000 Dijon, France; 5 Zoological Institute, University of Basel, 4051 Basel, Switzerland; 6 Department of Natural Sciences, Varaždin City Museum, 42000 Varaždin, Croatia; 7 Centre for Biodiversity Genomics, University of Guelph, Guelph, ON N1G 2W1 Canada

**Keywords:** Ecology, Evolution, Zoology

## Abstract

*Gammarus roeselii* Gervais, 1835 is a morphospecies with a wide distribution range in Europe. The Balkan Peninsula is known as an area of pre-Pleistocene cryptic diversification within this taxon, resulting in at least 13 Molecular Operational Taxonomic Units (MOTUs). The morphospecies diversified there during Neogene and has probably invaded other parts of the continent very recently, in postglacial or even historical times. Thus, the detailed goals of our study were to (1) identify which lineage(s) colonized Central-Western Europe (CWE), (2) determine their possible geographical origin, (3) verify, whether the colonisation was associated with demographic changes. In total, 663 individuals were sequenced for the cytochrome oxidase I (COI) barcoding fragment and 137 individuals for the internal transcribed spacer II (ITS2). We identified two MOTUs in the study area with contrasting Barcode Index Number and haplotype diversities. The Pannonian Basin (PB) appeared to be a potential ice age refugium for the species, while CWE was colonised by a single lineage (also present in PB), displaying low genetic diversity. Our results suggest that *G. roeselii* is a relatively recent coloniser in CWE, starting demographic expansion around 10 kya.

## Introduction

The current distribution of animal and plant species and their phylogeographic lineages have been largely shaped by the past geological and climatic events^1^. For example, the last Pleistocene glacial period and the subsequent climate warming, has had severe effects on the genetic diversity and distribution of numerous species in Europe^2^. At the time of the Last Glacial Maximum (LGM) at ca. 26–15 kya, the northern parts of the continent were covered by the Eurasian Ice Sheet Complex^3^. The cold temperature and the reduced water availability forced most of the local fauna and flora to retreat into isolated southern ice-age refugia. The most prominent were the three main peninsulas in the Mediterranean region: the Iberian, the Apennine and the Balkan Peninsula, where prolonged isolation led to the divergence of phylogeographic lineages^4^. The post-LGM climatic warm-up was associated with northward expansions often combined with a series of founder events, leading to a general geographic pattern of genetic diversity reduction from south to north in the case of many species^1,4,5^. Apart from the three main refugia, several extra-Mediterranean refugia have been recognized, e.g., the Alps, the Carpathian Arch, and the Pannonian Basin (PB)^6–10^.

The majority of studies on the topic of diversification associated with glacial refugia and postglacial colonizations focuses on terrestrial taxa^2, 6^. In freshwaters, the dynamics can be often different compared to terrestrial landscapes. In the former, the habitats could be either patchier and isolated, promoting divergence, or on the opposite, could be interconnected promoting exchange. For example, pre-Pleistocene tectonic movements and subsequent changes in watersheds, with isolated drainages, possibly impacted the rate of species diversification in many Eurasian freshwater taxa^11–16^. However, on the opposite, present distribution and population connectivity in freshwater fauna is largely affected by the postglacial climate warming, as well as by human impact^5,17–19^. The construction of large navigable waterways (canals, channels) has been a primary anthropogenic factor heavily affecting dispersal of species both in historical and recent times^20–22^.

Gammarid amphipods can be considered as challenging models for phylogeographic studies in fresh waters. Members of this group are permanently aquatic and have very limited ability to survive drought^23^. The natural dispersal abilities of amphipods between isolated water bodies are minimal; although recent studies documented local (small-scale) distribution of amphipod haplotypes across watersheds^24,25^. In addition, rare cases of ectozoochory by birds^26^ or by mammals^27^ are also documented. On the other hand, gammarids breed fast, as they usually have 1–3 generations per year^28^. Potentially, a few gravid females can establish a population^29^. Many species can survive adverse cold climatic conditions, including severe winters under ice cover^30^. For that reason, the location of their glacial refugia, timing of lineage diversification, and dynamics of post-glacial colonisation are hard to predict. On one end of the spectrum, old isolated water basins (e.g. ancient lakes) can hold endemic lineages and species^31–33^. Recent phylogeographic studies have indicated high molecular cryptic diversity within several amphipod morphospecies, with numerous Molecular Operational Taxonomic Units (MOTUs) representing phylogenetic species of pre-Pleistocene divergence^13,34^. This is especially true for morphospecies with wide geographic distribution ranges, such as *Gammarus balcanicus* Schäferna, 1923^13^ and *Gammarus fossarum* Koch, 1836^35^. On the other end of the spectrum, artificially connected large river systems allow for homogenization of freshwater fauna^21,36^ and mixing the genetic pools of previously isolated populations^37^. Fish restocking might also be a vector of amphipod introduction^38,39^. Amphipod crustaceans are listed among the richest in the number of invasive species successfully colonizing large water bodies (rivers and lakes) in Europe^40,41^. The prominent examples are *Dikerogammarus villosus* (Sowinsky, 1894)^40^, *Dikerogammarus haemobaphes*^42^ and *Pontogammarus robustoides* (G.O. Sars, 1894)^39^.

**Figure 1:**
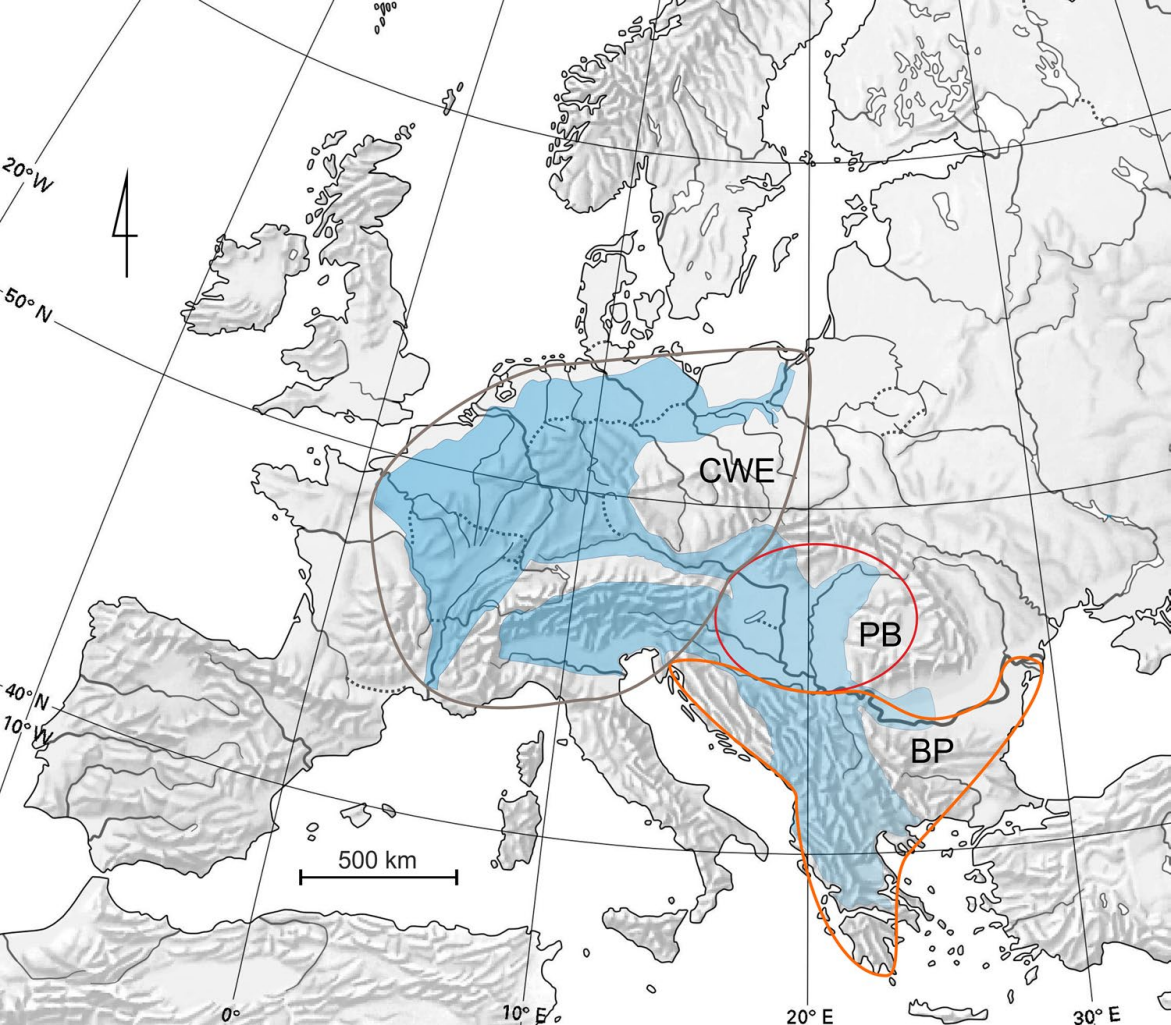
Geographic range of *Gammarus roeselii*. Rebuilt after Jażdżewski (1980)^20^, Jażdżewski & Roux (1988)^43^, Copilaş-Ciocianu et al.^96^ and our own data. CWE = Central-Western Europe, PB = Pannonian Basin, BP = Balkan Peninsula. Map created with QGIS 3.4.5 (https://www.qgis.org/fr/site/).

*Gammarus roeselii* has a wide but very peculiar distribution in Europe (Fig. 1). In the Balkans, *G. roeselii* can be found in various types of water bodies, from mountain streams, through rivers, to lakes^11^. Outside the Balkans, this species is associated mainly with the network of rivers and canals (Fig. 1). Already several decades ago, some studies suggested that *G. roeselii* originated from the Balkan Peninsula and its expansion outside the Balkans i.e., into the Pannonian Basin (PB) and Central-Western Europe (CWE) took place in historical times and progressed through artificial canals, possibly with the unintentional help of humans transferring water plants^20,43^. Currently, in PB and CWE, *G. roeselii* is considered as an old, well-established alien species^17^. This alien-exotic status is reinforced by recent reports of further expansion in France^44^ and Italy^45^. Surprisingly, *G. roeselii* was described by Gervais in 1835 from Coulanges-sur-Yonne (*locus typicus*), located 200 km south-east of Paris on the Yonne river. At that time, the place was not connected to the western part of *G. roeselii's* distribution^46^, thus presence of the species in those waterbodies was explained by human translocation with aquatic plants^20^. Jażdżewski^20^, as well as Jażdżewski and Roux^43^ suggested that presence of *G. roeselii* in the Oder River in Poland may be a result of both (1) the eastward colonisation through the Oder-Spree and Oder-Havel canal systems and (2) crossing the rather low watershed between the Oder and the Morava River (a Danube tributary) in the Czech Republic. In addition, the authors did not exclude that part of the current distribution of *G. roeselii* outside the Balkans could be associated with a natural post-glacial expansion, possibly of a single lineage, especially in the lower part of the Danube basin. In agreement with this hypothesis, Moret and colleagues^47^, based on 16S mtDNA, as well as Copilaş-Ciocianu and colleagues^48^, based on COI, suggested that only one phylogeographic lineage is present in Hungary, Czech Republic, but also France. On the other hand, Grabowski et al.^11^ recently identified 13 pre-Pleistocene MOTUs in the Balkan Peninsula showing that *G. roeselii* is characterised by a high level of cryptic diversity within this area.

The aim of our present study was to examine the phylogeography of *G. roeselii* in PB and CWE in relation to the 13 pre-Pleistocene MOTUs recently identified by Grabowski et al.^11^ in the Balkan Peninsula. To complete this goal, we: (1) hypothesised that PB could host further cryptic diversity acting as an additional glacial refugium, (2) tested how many lineages are present in PB and if this area could have served as a source for the colonisation of CWE.

## Results

### Mt-DNA: COI haplotypes, BIN and MOTU definition, divergence and diversification time-frames

**Figure 2:**
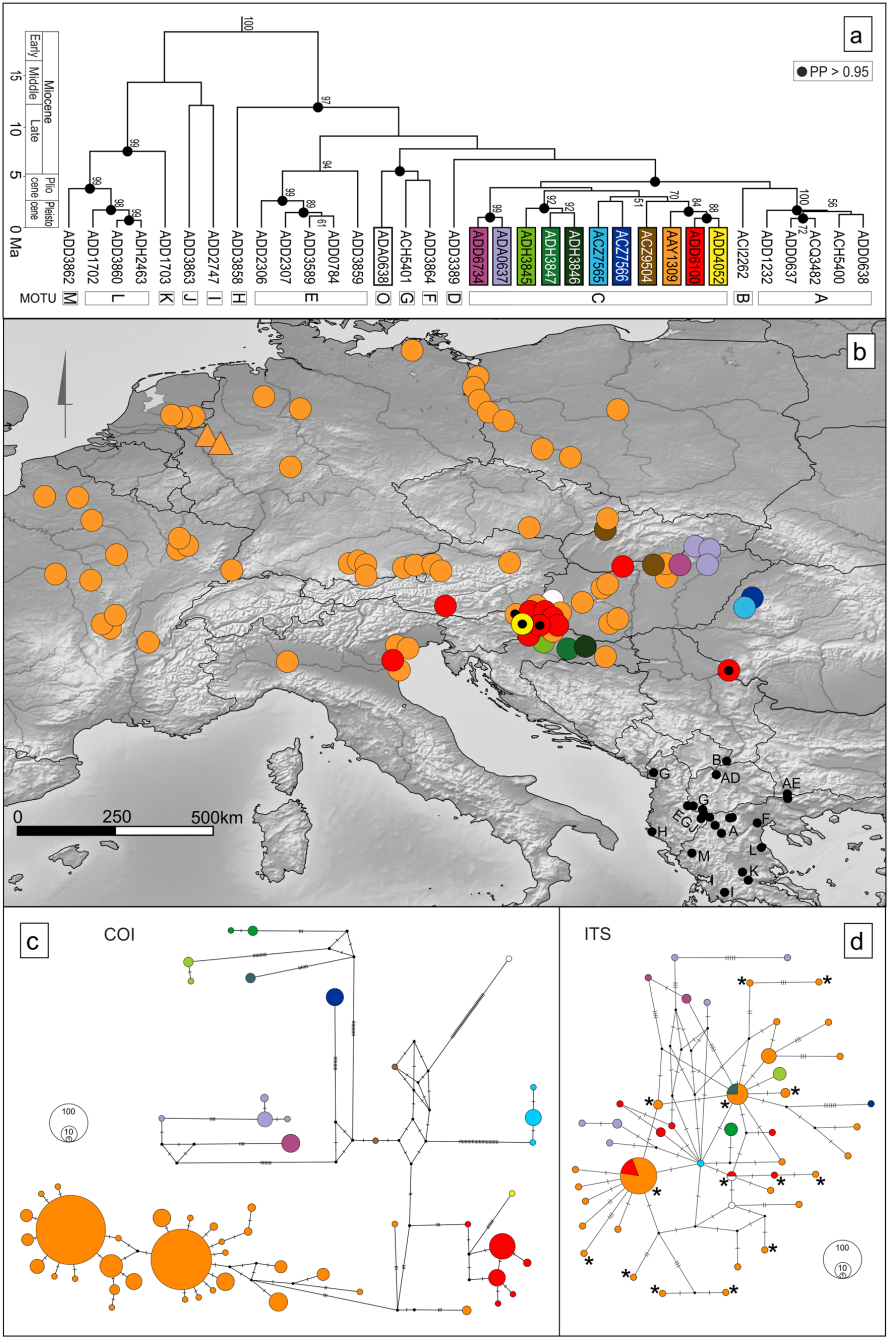
Phylogeny and distribution of *Gammarus roeselii* COI BINs: (**a**) Bayesian tree reconstructed from representative sequences of all COI BINs with MOTUs definition. MOTUs were defined after Grabowski et al.^11^. BINs produced in this study are indicated by frames. Values at nodes indicate the bootstrap values (above 0.5) for the parallelly reconstructed ML tree. (**b**) Map showing all sampling sites of the study. Colours indicate BINs of **A**. Triangles indicate sampling sites of sequences from Grabner et al.^49^. Black dots represent the sites from Grabowski et al.^11^, displaying also the COI MOTUs found in the Balkan Peninsula. (**c** & **d**) Haplotypic networks of COI and ITS2 markers, respectively. Colour codes indicate COI BINs. Stars on the ITS2 network indicate ITS2 haplotypes which were present in northern Italy. Map created with QGIS 3.4.5 (https://www.qgis.org/fr/site/).

Out of 711 individuals from the Pannonian Basin (PB) and the Central-Western Europe (CWE), 55 COI haplotypes were defined (Table 1; Fig. 2c) which were clustered in BOLD into twelve Barcode Index Numbers (BINs) (Fig. 2a,c). Three of these BINs (AAY1309, ADD4052, ADD6100) were known already from the four northernmost sites in the study by Grabowski et al.^11^, i.e., PB. Altogether, at the scale of Europe (including the Balkan Peninsula), a total of 34 BINs is now registered (Fig. 2a). The BI tree allowed to group these 34 BINs in 14 MOTUs (Fig. 2a), two of them, MOTUs O and C, being present in PB and only one, MOTU C, in CWE (Fig. 2b).

**Table 1:** Sampling of *Gammarus roeselii* and its molecular diversity.

Site no	Region	Country	Site	River basin	Lat (N)	Long (E)	Alt (m a.s.l.)	MOTU	BIN	N	COI	ITS2
1	PB	SK	Borcice	Danube	48.976	18.151	218	C	AAY1309	8	4(1) 6(2) 7(2) 8(2)	–
									*ACZ9504*		*5(1)*	*–*
2	PB	SK	Bina	Danube	47.918	18.646	116	C	AAY1309	3	7(2) 22(1)	–
									ADD6100	7	16(7)	2(2)
3	PB	HU	Szarvasgede	Danube	47.827	19.645	126	C	AAY1309	11	12(11)	2(2)
									ACZ9504	1	13(1)	–
4	PB	HU	Maconka stream	Danube	47.9952	19.8651	197	C	AAY1309	12	12(12)	2(1) 6–35(1)
5	PB	HU	Pusztavam	Danube	47.413	18.221	200	C	AAY1309	12	14(11) 12(1)	1(2)
6	PB	HU	Jasd	Danube	47.295	18.054	204	C	AAY1309	10	12(9) 15(1)	1(2)
7	PB	HU	Tapolca	Danube	46.8717	17.4382	114	C	AAY1309	8	12(1) 41(6) 42(1)	30(1)
8	PB	HU	Bikal stream	Danube	46.3335	18.2938	148	C	AAY1309	11	50(11)	26(1) 31–32(1)
9	PB	HU	Szalatnak	Danube	46.2877	18.2737	155	C	AAY1309	12	12(6) 50(5) 51(1)	1(1) 22(1)
10	PB	HR	Breznica (upstream)	Danube	45.3328	18.1559	196	C	AAY1309	3	60(3)	7(1) 45(1)
11	PB	RO	Makoviste	Danube	44.9425	21.661	143	C	ADD6100	12	16(12)	–
12	PB	HU	Kerkabarabas	Danube	46.678	16.565	172	C	AAY1309	7	12(6) 20(1)	–
									ADD6100	5	18(5)	–
								O	ADA0638	1	19(1)	17–18(1)
13	PB	HU	Paka	Danube	46.602	16.632	159	C	AAY1309	8	12(6) 17(2)	4(1)
14	PB	HU	Lispeszentadorjan-Kicsehi	Danube	46.53	16.683	146	C	AAY1309	6	12(6)	28–29(1) 1(1)
									ADD6100	4	16(4)	–
15	PB	HR	left canal of reservoir Dubrava (Donja Dubrava)	Danube	46.3219	16.7528	139	C	ADD6100	5	18(4) 62(1)	2(2) 40(1) 41(1) 41–42(1)
16	PB	HR	Gliboki potok (Apatovec)	Danube	46.1518	16.6007	207	C	AAY1309	6	59(6)	7(6)
17	PB	HR	Belica (Belica)	Danube	46.4109	16.5504	147	C	ADD6100	6	18(5) 63(1)	2(6)
18	PB	HR	Varaždin	Danube	46.319	16.359	170	C	ADD6100	1	18(1)	–
19	PB	HR	Belski potok downstream (Bela)	Danube	46.2067	16.2602	139	C	ADD6100	2	61(2)	43(1)
20	PB	Sl	Nova Vas	Danube	46.382	15.939	215	C	AAY1309	1	4(1)	–
									ADD4052	1	64(1)	–
21	PB	Sl	Creta	Danube	46.544	15.614	360	C	AAY1309	3	4(3)	–
22	CWE	CZ	near Pulkov	Danube	49.0308	15.9925	401	C	AAY1309	10	4(3) 12(7)	–
23	CWE	AT	Kirchberg	Danube	48.029	15.4331	367	C	AAY1309	11	12(1) 40(10)	2(2)
24	CWE	AT	Miltstattersee	Danube	46.789	13.611	600	C	ADD6100	12	16(11) 36(1)	2(2)
25	CWE	IT	Caposile	Piave	45.578	12.5508	1	C	AAY1309	10	12(10)	7(1) 8(1) 10–11(1)
26	CWE	IT	Porto Tolle	Po	44.9514	12.3031	5	C	AAY1309	3	12(3)	2(1)
27	CWE	IT	Brenta dAbba	Brenta	45.233	12.1139	2	C	ADD6100	2	18(2)	16–17(1)
28	CWE	IT	Treviso	Sile	45.6557	12.2195	10	C	AAY1309	12	12(12)	6(1) 7(2) 7–8(1) 12–13(1)
29	CWE	IT	Chiozzo	Po	45.1936	9.1214	61	C	AAY1309	12	12(12)	2–7(1) 14–15(1) 20–21(1)
30	CWE	AT	Attersee	Danube	47.79	13.487	480	C	AAY1309	7	4(1) 21(6)	2(2)
31	CWE	AT	Irrsee	Danube	47.892	13.316	560	C	AAY1309	12	1(5) 4(7)	2(1) 9(1)
32	CWE	DE	Wagingersee	Danube	47.954	12.749	455	C	AAY1309	11	1(9) 12(2)	2(1)
33	CWE	DE	Chiemsee	Danube	47.884	12.418	517	C	AAY1309	11	1(10) 32(1)	2(1)
34	CWE	DE	Kochelsee	Danube	47.657	11.356	620	C	AAY1309	12	12(12)	2(2)
35	CWE	DE	Starnbergersee	Danube	47.973	11.352	600	C	AAY1309	12	12(12)	2(1)
36	CWE	DE	Ammersee	Danube	48.077	11.134	530	C	AAY1309	12	1(4) 12(7) 33(1)	2(1)
37	CWE	DE	Pitzingsee	Danube	48.007	10.88	615	C	AAY1309	12	34(11) 35(1)	2(1)
38	CWE	FR	Pont de Cheny	Rhone	45.751	5.176	200	C	AAY1309	5	1 (5)	–
39	CWE	FR	Briennon	Atlantic	46.147	4.097	252	C	AAY1309	12	1 (12)	2 (2)
40	CWE	FR	Marcigny	Atlantic	46.277	4.016	245	C	AAY1309	12	1(12)	2(2)
41	CWE	FR	Volesvres	Loire	46.47	4.162	246	C	AAY1309	12	1(12)	2(2)
42	CWE	FR	Saint Gondon	Loire	47.6997	2.54222	132	C	AAY1309	5	1(3) 12(2)	2(1)
43	CWE	FR	Coulanges	Loire	47.524	3.541	149	C	AAY1309	10	1 (10)	–
44	CWE	FR	Lusigny-sur-Barse	Seine	48.253	4.272	116	C	AAY1309	5	12 (4), 37 (1)	2 (3)
45	CWE	FR	Epernay	Seine	49.236	3.572	196	C	AAY1309	1	1 (1)	2(1)
46	CWE	FR	Argoeuves	Somme	49.923	2.229	16	C	AAY1309	10	1 (3), 12 (7)	2 (3)
47	CWE	FR	Bihecourt	Somme	49.891	3.171	72	C	AAY1309	11	1 (1), 12 (10)	2 (2)
48	CWE	FR	Xeuilley	Rhine	48.576	6.108	223	C	AAY1309	12	1(12)	–
49	CWE	FR	Pierreville	Rhine	48.55	6.126	227	C	AAY1309	12	1(12)	2(1)
50	CWE	FR	Chalampe	Rhine	47.814	7.546	210	C	AAY1309	12	1(11) 48(1)	2(2)
51	CWE	NL	Schipbeek	Rhine	52.245	6.324	12	C	AAY1309	12	1(9) 12(2) 25(1)	2(2)
52	CWE	NL	Dortherhoek	Rhine	52.229	6.278	7	C	AAY1309	12	1(1) 12(9) 25(2)	2(1) 2–3(1)
53	CWE	NL	Twente Canal	Rhine	52.227	6.603	7	C	AAY1309	3	12(1) 25(2)	–
54	CWE	DE	Rapphoffs Mühlenbach	Rhine	51.6643	6.9763	29	C	AAY1309	15	1(15)	–
55	CWE	DE	Kemnader Lake	Rhine	51.4229	7.2679	71	C	AAY1309	15	1(13) 12(2)	–
56	CWE	DE	Goldenstedt	Weser	52.781	8.454	40	C	AAY1309	11	1(10) 3(1)	2(1) 36(1)
57	CWE	DE	Antrifsee	Rhine	50.766	9.21	284	C	AAY1309	12	1 (5), 38 (7)	2 (2)
58	CWE	DE	Wunstorf	Weser	52.433	9.489	42	C	AAY1309	12	1 (6), 12 (6)	2 (2)
59	CWE	DE	Bad Suelze	Recknitz	54.105	12.664	2	C	AAY1309	13	1 (13)	5(1)
60	CWE	PL	near Debogora	Oder	53.15	14.452	27	C	AAY1309	10	1(9) 12(1)	2(2)
61	CWE	PL	Zurawki	Oder	53.226	14.497	7	C	AAY1309	12	1(12)	2(1)
62	CWE	PL	near Reczyce	Oder	52.676	14.572	21	C	AAY1309	11	1(2) 12(8) 24(1)	2(3)
63	CWE	PL	near Rzepin	Oder	52.336	14.834	55	C	AAY1309	12	1(11) 23(1)	2(3)
64	CWE	PL	near Szklarka Rudnicka	Oder	52.089	15.272	50	C	AAY1309	10	1(4) 12(6)	2(2)
65	CWE	PL	Prochowice	Oder	51.279	16.3642	100	C	AAY1309	3	12(2) 39(1)	–
66	CWE	PL	Siechnice	Oder	51.0421	17.1589	120	C	AAY1309	12	4(12)	2(2)
67	CWE	PL	Lysek	Oder	52.4068	18.5036	89	C	AAY1309	14	1(14)	2(1)
68	PB	HU	Szin	Danube	48.497	20.69	164	C	ADA0637	5	11(5)	25(1)
69	PB	HU	Szamaszend	Danube	48.398	21.119	153	C	ADA0637	9	11(7) 30(1) 31(1)	23(2) 24(1)
70	PB	HU	Kesznyeten	Danube	47.966	21.05	114	C	ADA0637	3	11(1) 29(2)	27(1) 37–38(1)
71	PB	RO	Fughiu	Danube	47.059	22.0417	155	C	ACZ7566	10	9(10)	19(1)
72	PB	RO	Halta	Danube	46.998	22.003	173	C	ACZ7565	11	26(10) 27(1)	39(1)
73	PB	HR	L tributary of Voćinska rijeka (Vocin,1)	Danube	45.6236	17.5861	180	C	ADH3846	4	56(4)	7(6)
74	PB	HR	Velika Čavlovica (Dežanovac)	Danube	45.5589	17.0867	122	C	ADH3847	5	57(1) 58(4)	44(5)
75	PB	HR	Glogovnica (Grujice)	Danube	46.1329	16.5564	189	C	ADH3845	5	54(4) 55(1)	46(5)
76	PB	HU	Bator stream	Danube	47.9648	20.27	228	C	ADD6734	12	49(12)	34(1) 33–34(1)
77	CWE	FR	Puligny	Rhine	48.539	6.139	227	C	AAY1309	12	1(12)	2(1)

CWE = Central-Western Europe, PB = Pannonian Basin, MOTU = Molecular Operational Taxonomic Units. BINs = Barcode Index Numbers. COI and ITS2 = two genetic markers used in the present study with haplotype number followed in parenthesis by the number of individuals. Country codes follow ISO 3166-1 alpha-2—two-letter country codes: SK = Slovakia, HU = Hungary, HR = Croatia, RO = Romania, SI = Slovenia, CZ = Czechia, AT = Austria, IT = Italy, DE = Germany, FR = France, NL = The Netherlands, and PL = Poland.

MOTU O is new, includes only the BIN ADA0638, and it diverged in the late Miocene from its sister clade containing MOTUs F and G found in the northern part of the Balkan Peninsula (Fig. 2a,b).

MOTU C includes the three BINs from Grabowski et al.^11^, as well as eight new BINs (ADD6100, ACZ7565, ACZ7566, ADH3845, ADH3846, ADH3847, ADA0637 and ADD6734). The diversification of BINs within MOTU C occurred in the Pannonian Basin. It has started in the middle Pliocene and peaked during the Pleistocene. MOTU C is in a phylogenetic sister relationship with two MOTUs (A and B) found in the northern part of the Balkan Peninsula, from which it diverged in late Miocene (Fig. 2a,b, Supplementary Fig. S1).

### Detailed geographic distribution of haplotypes and BINs in CWE

Outside the Balkan Peninsula the relative abundance and distribution of the different BINs are highly variable. Ten BINs are rare (1 to 17 individuals) and not found outside the Pannonian Basin. BINs, such as ADH3845, ADH3846 and ADH3847 from northern Croatia, ACZ7565 and ACZ7566 from the Apuseni Mountains in Western Romania, as well as ADD6734 from the North Hungarian Mountains, are restricted to single localities. The BINs ADA0637 and ACZ9504 include individuals from several localities, but close to each other geographically (Fig. 2b).

The BIN ADD6100 is moderately frequent (56 individuals, 7.88%) and relatively widespread, occurring in the area extending from the Pannonian Basin, westwards to the Alps and to northern Italy (Fig. 2b).

The most frequent BIN is AAY1309 (583 individuals, 82%), being present in the Pannonian Basin and almost all around CWE, from the catchment of the Somme River in France, through the Netherlands and Germany, the Alps in Austria, Czech Republic, the basin of Po River in northern Italy, and western Poland, reaching the Vistula River on the east (Fig. 2b).

**Figure 3:**
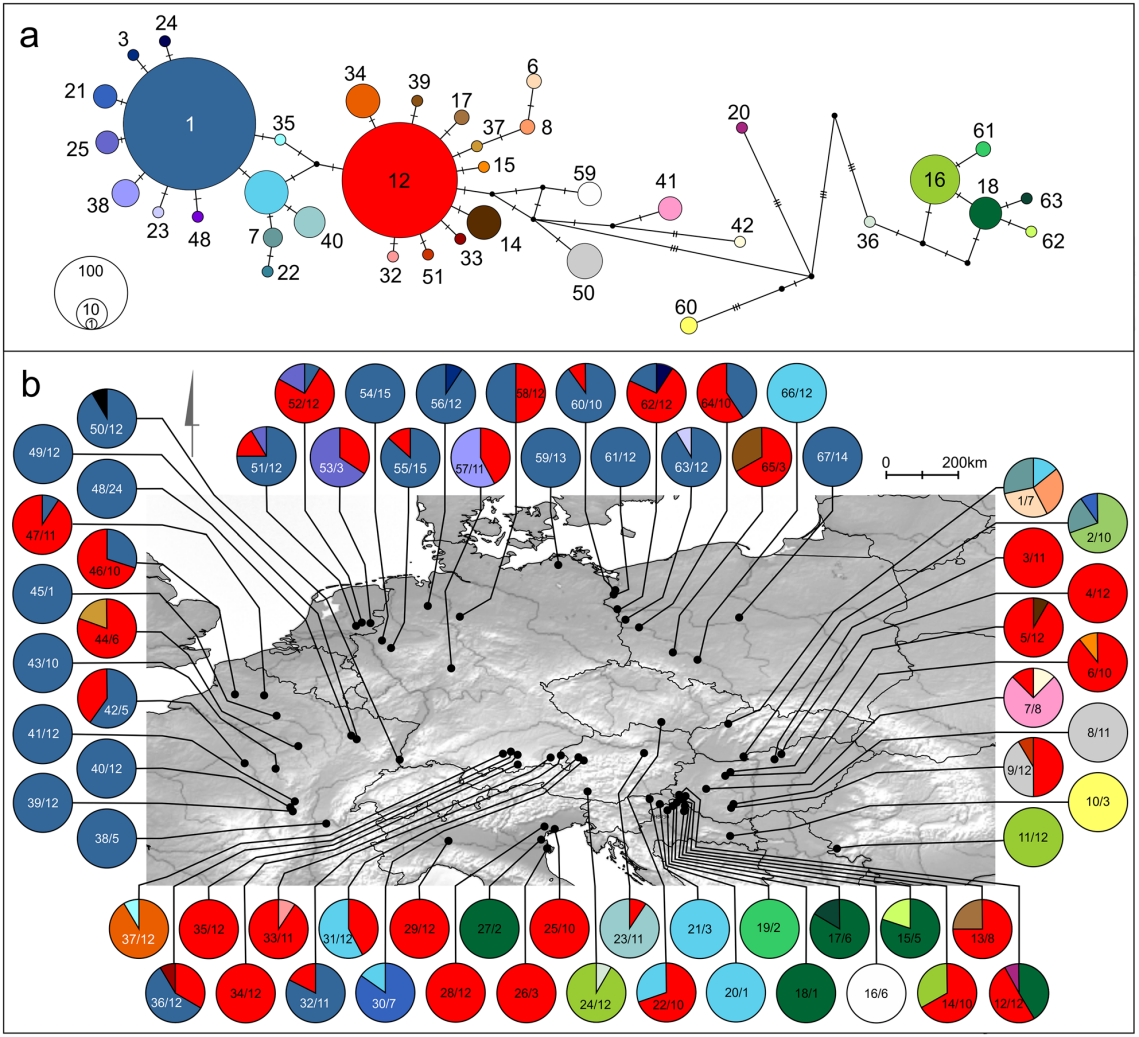
Haplotype distribution and frequencies of the two most widely distributed *Gammarus roeselii* COI BINs: (**a**) Haplotype network of BINAAY1309 and AAD6100. (**b**) Distribution and frequencies of haplotypes. Pie charts represent single localities. Site number (as in Table 1) and number of sequenced individuals per site are indicated. Colour codes for haplotypes are based on the haplotype network of the two BINs. Map created with QGIS 3.4.5 (https://www.qgis.org/fr/site/).

The MJ haplotype network shows the presence of 6 haplotypes within BIN ADD6100, while BIN AAY1309 is more diverse, including 31 haplotypes, with higher genetic distances between them (Fig. 3a). BINs AAY1309 and ADD6100 co-occur only on three sites in the Pannonian Basin (Fig. 3b; Table 1). BIN ADD6100 is characterised by a higher haplotype diversity in the Pannonian Basin compared to northern Italy. Within BIN AAY1309, the most frequent haplotype, haplotype 1, and the rare peripheral haplotypes are generally frequent in the northern and western parts of CWE, while haplotype 12 and the surrounding haplotypes can be found more often in the central and southern areas (Fig. 3b). Some sites from the Pannonian Basin contain private and divergent haplotypes of BIN AAY1309 (haplotype 20, haplotype 60).

### Mitochondrial versus nuclear haplotype relationships

The phylogenetic relationships based on COI and ITS2 haplotypes are illustrated with MJ haplotype networks (Fig. 2c,d).

In the COI network, the two most widely distributed BINs AAY1309 and AAD6100 consist, as already pointed out, of the highest number of haplotypes and individuals. Other BINs that have more localized distribution hold fewer haplotypes and are represented by peripheral haplotypes in the MJ network. BIN ADA0638, which forms MOTU O, is represented by only one haplotype, extremely divergent from all the others (Fig. 2c), as expected given its position in the BI tree (Fig. 2a).

The overall topology of the ITS2 network is largely incongruent with the COI-based one (Fig. 2d). As expected, the genetic distances between ITS2 haplotypes are smaller than in case of COI^49,50^, but at the same time, the topology and phylogenetic relationships are more complex. There is one haplotype represented by the highest number of individuals (ITS2 haplotype 2), while most of the other haplotypes are unique to single individuals (Fig. 2d). The grouping of COI haplotypes into BINs is not well reflected in the ITS2 network. Concerning MOTU C, the BINs AAY1309 and AAD6100 share the most frequent ITS2 haplotype (haplotype 2), while other ITS2 haplotypes corresponding to these BINs are scattered all around the network. Also the BINs AAY1309 and ADH3846 share one haplotype. Interestingly, MOTU O (BIN ADA0638) is characterized by one unique COI haplotype (haplotype 18) but its ITS2 haplotype is commonly observed and associated with an individual assigned to BIN AAY1309 of MOTU C. On the other hand, individuals of BIN ADA0637 are parted away from each other in the ITS2 network, while based on the COI sequences they are closely related. Only the individuals of the BINs ADA0637, ADD6734, ACZ7566, highly divergent on the COI network, show also the highest level of divergence on the ITS2 network. On the contrary, the BINs ADA0638 and ACZ7565 represent distant groups in the COI network, while in the ITS2 network both of these BINs have a central position, with few mutations separating them from the most frequent haplotype.

An interesting pattern can be observed while comparing the geographic distribution of the COI and ITS2 haplotypes to their position in the MJ networks. While in the case of COI, distant peripheral haplotypes are present only in the Pannonian Basin, the ITS2 haplotypes of the corresponding individuals are present both in the Pannonian Basin and in northern Italy. There are some relatively divergent ITS2 haplotypes observed only in northern Italy. These haplotypes can be found in different parts of the network, not forming a well-defined subgroup. At the same time, the COI haplotypes appearing in northern Italy are not unique to this region. One is the widely distributed haplotype 12, and the other one is haplotype 18, which also occurs in the Pannonian Basin (Table 1).

### Demographic history

Although not univocal, the results of mismatch distribution analyses do not reject the scenario assuming demographic and spatial expansions for BINs AAY1309 and ADD6100 (Table 2; Supplementary Fig. S2). In the case of BIN AAY1309 a peak in the observed data can be detected at three pairwise differences. It is associated with the fact that this BIN contains two haplotypes (haplotype 1 and 12), each with a large number of individuals, divergent by three substitutions (Fig. 3a). The values of Tajima’s D neutrality test do not support the existence of population expansion of BIN AAY1309 neither in CWE, nor in the PB (Table 2). Fu’s F supports the expansion in CWE, but not in the PB (Table 2). In the case of BIN ADD6100 the values of both neutrality tests are negative, although non-significant (Table 2).

**Table 2:** Results of demographic analyses for the two most widely distributed BINs in CWE, AAY 1309 and ADD6100.

	AAY1309				ADD6100 (n = 56)	
	CWE (n = 420)		PB (n = 126)			
	Demographic expansion	Spatial expansion	Demographic expansion	Spatial expansion	Demographic expansion	Spatial expansion
τ	3.484	2.086	3.879	2.966	0.773	0.77
SSD	0.029	0.262	0.034	0.019	0.01	0.01
SSD *p* value	0.41	0.38	0.028	0.61	0.14	0.04
Hr index	0.095	0.095	0.094	0.094	0.13	0.13
Hr index *p* value	0.41	0.58	0.26	0.61	0.15	0.14
Tajima’s D	− 1.0804		− 0.77537		− 0.914	
Tajima’s D *p* value	0.118		0.203		0.207	
Fu’s F	− 7.48343		− 2.88168		− 2.169	
Fu’s F *p* value	0.031		0.164		0.098	

Parameters of the mismatch distribution analyses and results of Tajima’s D and Fu’s F neutrality tests are presented. τ = Tau; SSD = Sum of Squared Deviation; Hr = Harpending’s Raggedness.

**Figure 4:**
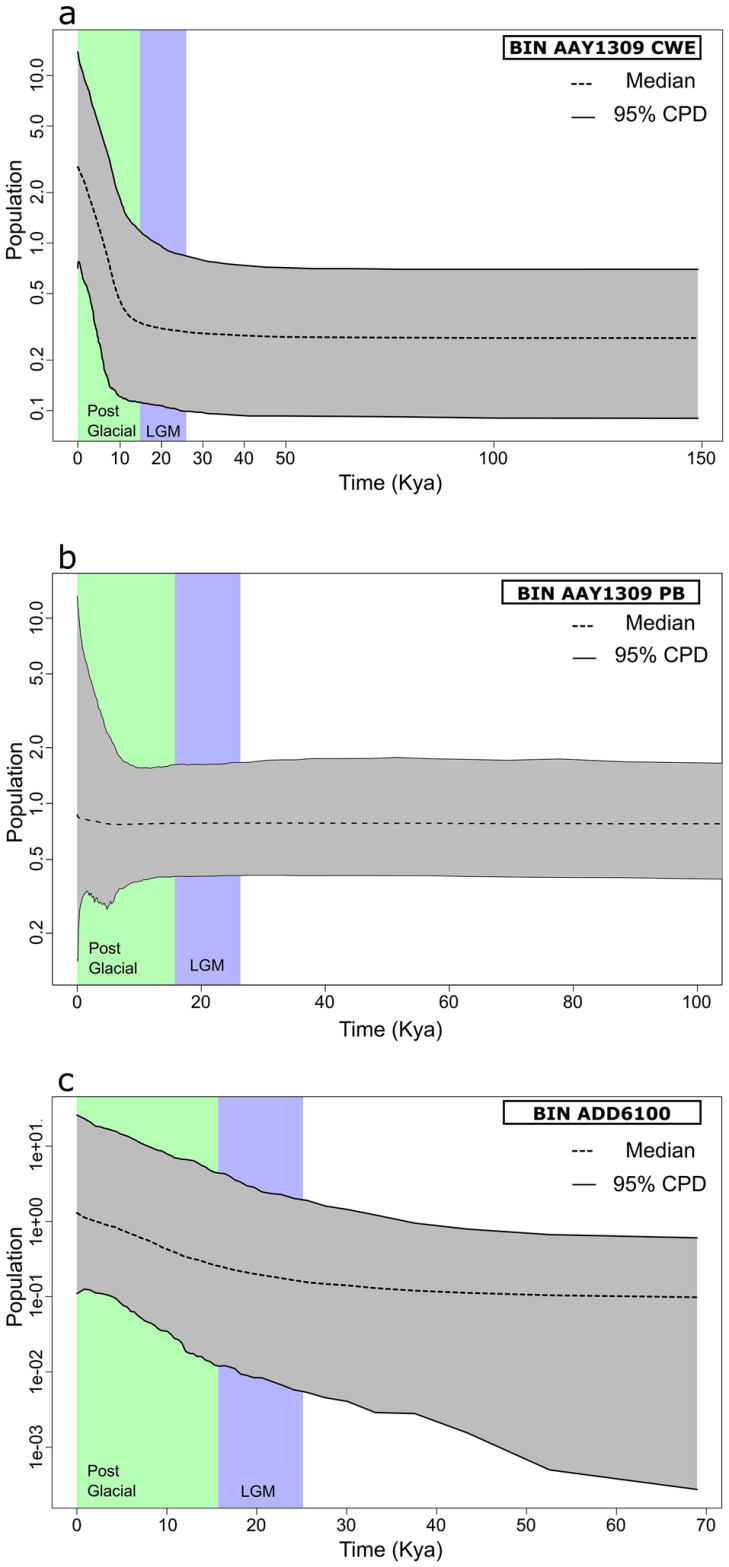
Extended Bayesian Skyline Plots of the two most widely distributed COI BINs the study area: (**a**) BIN AAY1309 in CWE (**b**) BIN AAY1309 in PB and (**c**) ADD6100. The plots show the relative variation of effective population size in time in Ky. Elevation on graphs indicates population expansion. CWE = Central Western Europe; PB = Pannonian Basin. EBSP were generated using BEAST 2.4.8 (https://www.beast2.org/) and visualized using R software (https://www.r-project.org).

The results of the EBSP analysis based on well represented COI dataset also show different demographic histories according to the two aforementioned BINs and/or regions (Fig. 4). The EBSP analyses for BIN AAY1309 support demographic growth of population starting ca. 10 Ka (Fig. 4a) for CWE, but not for PB (Fig. 4b). Considering BIN ADD6100, a slight increase of the population size in the last 10K years can be seen (Fig. 4c). The COI + ITS2 dataset supports these results however, in case of AAY1309 from CWE the expansion starts ca. 20 kya (Supplementary Fig. S3).

## Discussion

The goal of the present study was to reveal the phylogeographic history within the CWE and PB part of the distribution of freshwater amphipod *Gammarus roeselii.* Although the study was centred on CWE and PB, it also included the Balkan Peninsula covering the whole distribution range of the species.

In our study, based on the samples from CWE and PB, we defined two COI MOTUs i.e. MOTU C (already identified in Grabowski et al.^11^) and a new MOTU, MOTU O. In contrast, Grabowski et al.^11^ found 13 COI MOTUs just in the Balkan Peninsula. MOTU C is a sister clade to MOTUs A and B, while MOTU O is a sister clade to MOTU F and G. The common ancestor of both groups of MOTUs existed most probably in the early Pliocene, i.e. ca. 5 Mya (Fig. 2a). It supports the hypothesis that *G. roeselii* originated and diversified predominantly in the Balkan Peninsula. The presence of other such old and divergent local lineages was not detected in any geographical region north of the Balkans. These findings, therefore, support the hypothesis by Jażdżewski and Roux^43^, suggesting the Balkan Peninsula as the possible area of origin for the species. *G. roeselii* diverged here in the early Miocene mostly due to dynamic geological changes of the region and following habitat isolation; the topic was thoroughly discussed by Grabowski et al.^11^.

The diversity observed in the CWE and PB area was low on the COI MOTU level (only two MOTUs). Within each MOTU, the level of BIN diversity was highly contrasted. MOTU O included only one BIN and was restricted to one location in Hungary. On the opposite, we detected 11 BINs in MOTU C. Based on our results, most of the BINs appeared during the last 2.5 My, in Pleistocene. While the Pannonian Basin (PB) harbours all the BINs’ diversity present within MOTU C, the northern part of the Apennine Peninsula was colonised by only two BINs AAY1309 and ADD6100 (Fig. 2b), and the rest of CWE was colonized exclusively by the BIN AAY1309 (Fig. 2b). This is in agreement with the results of another study, which identified only one phylogeographic lineage based on 16S mtDNA data from Hungary, Czech Republic and France^47^.

The fact that the PB contains most BIN diversity within MOTU C, suggests that the region could also be, following the Balkans, an area of lineage diversification. Contemporarily with the diversification of the Balkan MOTUs, the area of the PB was covered by the brackish Pannonian Lake. The lake had completely disappeared by the end of Pliocene, i.e. ca. 2.5 Ma^51^, and the newly formed hydrological network could be colonised by *G. roeselii*, which subsequently diversified into the observed BINs.

The distribution of genetic diversity in the CWE seems to be consistent with the postglacial pattern proposed by Hewitt^1,2,52^. At the end of the LGM after the ice retreated, numerous species belonging to a variety of taxa, such as insects, mammals or fishes, were documented to (re)colonize the newly available areas from southern ice age refugia. Several fishes have colonised post-glacial Europe migrating up the Danube, crossing the, yet unstable, Danube/Rhine watershed and expanding to the just forming river systems of CWE (*ibidem*). Such post-glacial (re)colonization by a single lineage (the so called “leading-edge colonization”), as e.g. in the case of the meadow grasshopper (*Chorthippus parallelus* (Zetterstedt, 1821)), results in relative genetic uniformity over vast areas in the recently ice-freed northern regions, if compared to the southern source populations, often with much smaller ranges^1^. Recently, such a pattern was evidenced for the European freshwater crustaceans, such as the waterlouse, *Asellus aquaticus* (Linnaeus, 1758)^5^, and the scud, *Gammarus jazdzewskii*^53^. In the case of *G. roeselii*, an overall loss of genetic diversity can be recognized in the northern regions of the continent. Population inhabiting CWE consists of individuals belonging to a single lineage (BIN AAY1309), while the PB hosts higher diversity in the much smaller geographic area (Fig. 2b). In this context, and considering the previously discussed levels of diversity, we propose the PB as a potential extra-Mediterranean ice age refugium for *G. roeselii*. The PB was free of ice during the LGM and had a rich and complex hydrological network^3^. Some studies have already reported the region to hold refugial populations of terrestrial and freshwater organisms during the last glacial maximum [See^6^ for a review, but also^7, 8,10,54,55^]. The results presented here strongly suggest that only one lineage (BIN AAY1309) was successful in expanding from the PB refugium after the ice-sheet retreated over the North European Plain, the Alps, the western part of France. Both, the star-like topology of the COI haplotype network (Fig. 2c) and the results of mismatch distribution analysis (Supplementary Fig. S2; Table 2), point out that the population of *G. roeselii* in CWE (excluding Italy) could have experienced a recent demographic and spatial expansion. The EBSP analysis suggests such expansion could start at ca. 10 Kya in this area (Fig. 4a). During this time the transition between the late Younger Dryas and the early Holocene was taking place. The Eurasian Ice Sheet complex had fully retreated from the CWE region and the climate had begun to warm up steadily^56^. From the hydrological point of view this era was characterised by an increased groundwater supply, followed by the general stabilisation of floodplains of rivers^57,58^. Although there are well identified climate attributes of this transition period, the Holocene hydrological systems has also been affected by human activity (e.g. animal and plant domestication, and the increasing influence of agricultural practices)^59^.

We also cannot exclude the possibility of anthropogenic factors playing a role in the dispersion of *G. roeselii* in more recent, historical times. The species was first recorded and taxonomically described from the vicinity of Paris by Gervais in 1835. Jażdżewski^20^, as well as Jażdżewski and Roux^43^ hypothesized that *G. roeselii* dispersed to this area from the middle Danube basin, with unintentional human help, possibly via the system of artificial canals built across various watersheds or with traded water plants. Concerning the artificial waterways, the possible dispersal route could be upstream the Danube, then through the Ludwig canal between the Danube and Rhine drainage systems, and then through the rich canal network in France. The importance of artificial canals for aquatic species dispersal has been stressed in many studies and for numerous alien and invasive species in Europe^60^. In addition, recent expansion, i.e. over the last fifty years, at the edge of the distribution was documented through temporal faunistic surveys in France^20,43,44^ but also, Italy^45^ and Poland^61^. The expansion of the species to smaller tributaries and upper reaches of rivers has been also recently reported in Germany^62^.

The past success of *G. roeselii* to invade CWE as well as the ongoing upstream expansion can be promoted by some important ecological attributes of the species. Generally, *G. roeselii* shows high phenotypic plasticity to a variety of biotic (population density), abiotic (e.g. temperature, flow velocity), and anthropogenic (thermally polluted water influx) factors^62^. An elevated tolerance to anthropogenic impact was reflected in the species’ habitat preference in comparison to *G. fossarum*^63^. At the same time, the high adaptive potential of the species is expressed through its ability to coexist with other native gammarids (e.g. *G. pulex*)^64^. Moreover, in thermal conditions specific to the middle and lower sections of the rivers, which are classified as summer-warm running waters, it is able to outcompete some native species such as *G. fossarum*^65–68^ or *Echinogammarus stammeri* (S. Karaman, 1931)^45^. The morphological variation of the dorsal spines was also documented as a possible adaptation to predation avoidance^48^. Such traits might very well facilitate the past and present expansion of *G. roeselii*. Although no direct evidence exists, considering the Balkan origin of the species, the present day climate warming may also facilitate the species’ expansion.

The decreased ITS2 polymorphism and the overall lower divergence compared to COI can be explained by a lower mutation rate for rDNA. This phenomenon was reported for other invertebrates, such as marine gastropods^69^ and the holoplanktonic scyphozoan *Pelagia noctiluca* (Forsskål, 1775)^49^*.* Intra-individual polymorphism of ITS2, observed in our dataset, was also reported in other arthropods^70–72^. However, the most striking and interesting feature while comparing ITS2 and COI haplotype networks was the incongruences between their topologies. Interestingly, a little pool of relatively divergent ITS2 haplotypes is restricted to the Po River basin in the northern Apennine Peninsula while others are shared between the Po basin and the PB (Fig. 2b and stars on 2d). In comparison, the only two COI haplotypes present in the Apennine Peninsula are those most common and of widest distribution in Europe. One possible scenario explaining this pattern could be that *G. roeselii* colonised the northern Apennine Peninsula from the PB, where they got into secondary contact with a local population, most probably originating from earlier colonisation event(s). Similar mixing between lineages through historical headwater river captures has been observed in freshwater fish, which share habitat preferences with *G. roeselii,* such as *Phoxinus lumaireul* (Schinz, 1840)^73,74^, *Cobitis bilineata* Canestrini, 1865 and *Sabanejewia larvata* (De Filippi, 1859)^75^. Apparently, the contact of different lineages of *G. roeselii* resulted in an asymmetric introgression that eventually wiped out the local maternal lineages. Similarly, the fact that MOTU O (BIN ADA0638) is characterized by one unique COI divergent haplotype (haplotype 18) but its ITS2 haplotype is commonly observed and associated with individual assigned to BIN AAY1309 of MOTU C could be explained by such asymmetric mating. Such a pattern was also suspected to have taken place in the Balkan Peninsula^11^. In both cases, it is hard to explain why the local females have not contributed to the present genetic pool of the Apennine population. Directional mate-choice has been mostly studied in vertebrates, although this phenomenon is commonly observed also in invertebrate reproduction. In case of the waterlouse *Asellus aquaticus*, males (which are generally bigger than females) favour females with larger body size. Such larger females were shown to ovulate faster and produce bigger broods^76^. In the case of *Gammarus pulex* assortative mating was evident, with significant and strong selection on male body size^77^. At the same time the phenomenon was proven to be influenced by the female moulting stage, increasing in magnitude with females closer to moulting^78^.

## Conclusions

Our results supported the hypothesis that the Pannonian Basin was an important extra-Mediterranean glacial refugium for *Gammarus roeselii* and its divergence hotspot since the Pliocene. We also showed that *G. roeselii* MOTU C, and more precisely one BIN within this MOTU (BIN AAY1309), left the PB after the last glaciation, to colonise the rest of Central and Western Europe. We found also that the expansion of the *G. roeselii* MOTU C could have started already in the early Holocene and, as its distribution and genetic diversity patterns suggest, progressed in historical times aided by the construction of artificial waterways all over Europe. Finally, we presented an evidence suggesting asymmetric introgression events, which might be more common between invertebrate taxa, than previously thought.

## Materials and methods

### Overview of sampling strategy and dataset content

The present study focuses on the distribution of *G. roeselii* outside the Balkans, including 71 newly sampled localities as well as six localities from literature, i.e. four northernmost sites in the Pannonian Basin from Grabowski et al.^11^ and two sites in Germany from Grabner et al.^79^. All other sites located in the Balkans from Grabowski et al. 2017 were used to establish connections with phylogeographic lineages observed there^11^.

### Sampling and taxonomic identification of new individuals

Individuals of *Gammarus roeselii* were collected at 71 localities during sampling campaigns in the years 2005–2017, covering the whole species range outside the Balkans (Figs. 1, 2b). The sampling included streams, rivers and lakes. Samples were collected with benthic hand nets via kick and sweep method^61^. The collected material was sorted at a site, and gammarids were immediately fixed in 96% ethanol. Individuals were identified to the species level under stereomicroscope, using the taxonomic keys^46, 80,81^. All animals have been stored in the permanent collection of the Department of Invertebrate Zoology and Hydrobiology, University of Lodz, Poland.

### Laboratory procedures: DNA extraction, amplification and sequencing

DNA was extracted using the standard phenol–chloroform method^82^ from the muscle tissue of each individual from the newly sampled sites (1–12 individuals per site, altogether 663 individuals) (Table 1). The ca. 710 bp long part of the mitochondrial cytochrome oxidase I-subunit (COI) was amplified. Conditions of polymerase chain reaction (PCR) for the amplification of COI were set according to Grabowski et al.^11^. Following the definition of COI MOTUs and their geographic distribution (Fig. 2b; Table 1), representative individuals were chosen for amplification of the ca. 850 bp of the internal transcribed spacer 2 of the nuclear ribosomal operon (ITS2). PCR conditions for the amplification of ITS2 were set as following: pre-denaturation at 94 °C for 1 min followed by 35 cycles of denaturation at 94 °C for 30 s, annealing at 53 °C for 30 s, extension at 72 °C for 1 min 30 s, and final extension at 72 °C for 1 min 45 s (all primers used for amplification are specified in Supplementary Table S1). Prior to sequencing, the PCR products were cleaned using the EXO-FastAP method (Thermo Scientific). Sequencing was completed by Macrogen Europe—Amsterdam, The Netherlands, using BigDye terminator technology.

### Assembling the dataset for further analysis: sequence edition and alignment

Altogether, within this study, we sequenced 663 individuals for COI and 137 individuals for ITS2 (Table 1). The identity of all the sequences was verified with the BLASTn tool (https://blast.ncbi.nlm.nih.gov/blast)^83^. Raw sequences were trimmed, edited and aligned using Geneious 11.1.5 software (https://www.geneious.com/)^84^. The translation frame in COI sequences containing no stop codons was found. Eighteen sequences from Grabowski et al.^11^ and 30 sequences from Grabner et al.^79^ supplemented the COI dataset, leading to a total of 711 sequences (Table 1). Due to the presence of polymorphic copies of ITS2, the chromatograms of sequences from some individuals contained double peaks. Ambiguous sites were selected and coded with IUPAC ambiguity code in Sequencher 4 (https://www.genecodes.com/)^85^. Phasing and reconstruction of the two alleles for each individual were completed in DnaSP 6 (https://www.ub.edu/dnasp/)^86^. Haplotypes for both markers were identified by DnaSP 6 software. All the new sequences have been deposited in GenBank: COI: MT324702–MT325364; ITS2: MT325365–MT325501. Relevant voucher information for 663 individuals is accessible through the public dataset “DS-GROEEUR” (10.5883/DS-GROEEUR) in the Barcode of Life Data Systems (BOLD; https://v4.boldsystems.org).

### BIN and MOTU delimitation

Clustering sequences into BINs was performed in BOLD (https://v4.boldsystems.org), where every uploaded sequence was compared to each other and to already available records and got assigned to an existing or a newly created Barcode Index Number (BIN)^87^. BINs are registered in BOLD and are unique identifiers of sequence clusters, which are created based on a graph analytical distance-based approach aiming to find discontinuities between groups^87^.

The MOTU framework was taken from the Grabowski et al.^11^ paper, to keep reference to our previous study dealing with the Balkan diversification hotspot. In the present study we extended the dataset by adding more sequences from PB and from CWE. In result, with the exception of one, all the new sequences extended the clade which we referred to as MOTU C (see next paragraph for the phylogeny reconstruction). This MOTU has become more diverse than in Grabowski et al.^11^ but, still, it holds its integrity against its sister clade, composed of MOTUs A and B. The remaining sequence diverged in the late Miocene from its sister clade containing MOTUs F and G (see below), thus decided to name it as another MOTU, MOTU O, more affiliated to the Balkan MOTUs than to MOTU C.

### Time calibrated reconstruction of phylogeny

The time-calibrated phylogeny was reconstructed based on the COI sequences, using the Bayesian Inference (BI) in BEAST 2.4.8 (https://www.beast2.org/)^88^. One individual chosen at random from every BIN was used to build the tree (see previous paragraph for BIN definition). *Dikerogammarus villosus* (EF570297) and *Pontogammarus robustoides* (JF965990) sequences from GenBank (https://www.ncbi.nlm.nih.gov/genbank/) were used as an outgroup. The calibration points for the molecular clock were set the same as in the cross-validated calibration in Grabowski et al.^11^, with the initial rate set on 0.0113 substitutions per site per million years (My). The optimal model of evolution was selected using bModeltest (https://github.com/BEAST2-Dev/bModelTest/)^89^, clock model and tree prior were selected with the path sampling method in BEAST 2.4.8. The Tamura and Nei 1993 (TN93) model of nucleotide substitution with gamma-distributed rate heterogeneity (G) and a proportion of invariable sites (I) was chosen as the best-fitting model. The birth–death speciation process and lognormal relaxed molecular clock, were set as priors following Marginal Likelihood estimation through path sampling. The estimation was performed using BEAST software, the models were better for over 30 Bayes Factors in comparison to the next best-fitting model. Four MCMC runs were performed, each with 20 M iterations, sampled every 2000 iterations. The Effective Sampling Size (ESS) of each parameter was verified to be above 200 in Tracer 1.6 (https://beast.community/tracer)^90^. The outcomes of these runs were combined for tree construction in LogCombiner 2.4.8^88^ with the first 25% burn-in iterations discarded. After summarizing all sampled trees, the produced maximum clade credibility tree (MCC) was annotated in TreeAnnotator 2.4.8^88^, both programs being part of BEAST 2.4.8 package^88^ .

A maximum likelihood (ML) tree was constructed on the same dataset in MEGA7 software^91^ to give additional support to the topology of the Bayesian chronogram. The same model of evolution (TN93 + G + I) as in case of BI was used. To assess topology robustness 1000 bootstrap replicates were applied. These bootstrap values were reported on the chronogram (Fig. 2a).

### Historical demography: haplotype diversity and distribution, population expansion

For COI and ITS2 median-joining (MJ) haplotypic networks were generated in PopArt 1.7 (https://popart.otago.ac.nz/index.shtml)^92^. The homoplasy level parameter was set to the default value (ε = 0). Networks were colour-coded based on the haplotypes belonging to BINs provided by BOLD.

The demography of the two most widely distributed BINs (BIN AAY1309 and ADD6100, see Results) was investigated. COI haplotype 20 and 60 were removed from the dataset, due to their divergent position on the haplotype network (Fig. 3a). Individuals from northern Italy were also removed due to the possible presence of introgression (see below) and geographical distinctness. Two BINs were run separately, additionally BIN AAY1309 was separated in two populations, first containing the individuals from CWE and the second containing those from PB. First, historical demographic and spatial expansion were examined by mismatch analyses. Additionally, the demographic expansion was tested with Tajima’s D^93^, and Fu’s F^94^ neutrality tests. All the analyses were performed in Arlequin 3.5.1.3 (https://cmpg.unibe.ch/software/arlequin35/)^95^. Finally, to infer the changes in the effective population size over time, Extended Bayesian Skyline Plot (EBSP) was generated in BEAST 2.4.8^88^. The rate of 0.0113 substitution per site per My was used for COI. The preferred models of evolution for the aforementioned two BINs were set up with bModeltest^89^. EBSP was run separately for COI and COI + ITS2 datasets. The population scaling factor was set to 0.5 for COI and 2.0 for ITS2. Each run was 40 M MCMC long, sampled every 20,000 iterations. The ESS of each parameter was verified to be above 200 in Tracer 1.6. The plots were generated after removal of 10% burn-in using R software (https://www.r-project.org).

## Supplementary information

Supplementary Information

## Data Availability

All sequences with metadata are available in BOLD dataset DS-GROEEUR (10.5883/DS-GROEEUR), GenBank accessions numbers for COI: (MT324702-MT325364) and ITS: (MT325365-MT325501).

## References

[1] Hewitt GM (1996). Some genetic consequences of ice ages, and their role in divergence and speciation. Biol. J. Linn. Soc..

[2] Hewitt GM (2004). Genetic consequences of climatic oscillations in the quaternary. Philos. Trans. R. Soc. B Biol. Sci..

[3] Patton H (2017). Deglaciation of the Eurasian ice sheet complex. Quat. Sci. Rev..

[4] Hewitt GM (1999). Postglacial re-colonisation of European biota. Biol. J. Linn. Soc..

[5] Sworobowicz L (2015). Revisiting the phylogeography of *Asellus aquaticus* in Europe: insights into cryptic diversity and spatiotemporal diversification. Freshw. Biol..

[6] Schmitt T, Varga Z (2012). Extra-Mediterranean refugia: the rule and not the exception?. Front. Zool..

[7] Verovnik R, Sket B, Trontelj P (2005). The colonization of Europe by the freshwater crustacean *Asellus aquaticus* (Crustacea: Isopoda) proceeded from ancient refugia and was directed by habitat connectivity. Mol. Ecol..

[8] Sworobowicz L, Mamos T, Grabowski M, Wysocka A (2020). Lasting through the ice age: the role of the proglacial refugia in the maintenance of genetic diversity, population growth, and high dispersal rate in a widespread freshwater crustacean. Freshw. Biol..

[9] Neumann K (2005). Genetic spatial structure of European common hamsters (*Cricetus cricetus*)—a result of repeated range expansion and demographic bottlenecks. Mol. Ecol..

[10] Fussi B, Lexer C, Heinze B (2010). Phylogeography of *Populus alba* (L.) and *Populus tremula* (L.) in Central Europe: secondary contact and hybridisation during recolonisation from disconnected refugia. Tree Genet. Genomes.

[11] Grabowski M, Mamos T, Bącela-Spychalska K, Rewicz T, Wattier RA (2017). Neogene paleogeography provides context for understanding the origin and spatial distribution of cryptic diversity in a widespread Balkan freshwater amphipod. PeerJ.

[12] Hou Z, Sket B, Fiser C, Li S (2011). Eocene habitat shift from saline to freshwater promoted Tethyan amphipod diversification. Proc. Natl. Acad. Sci..

[13] Mamos T, Wattier R, Burzyński A, Grabowski M (2016). The legacy of a vanished sea: a high level of diversification within a European freshwater amphipod species complex driven by 15 My of Paratethys regression. Mol. Ecol..

[14] Perea S (2010). Phylogenetic relationships and biogeographical patterns in Circum-Mediterranean subfamily Leuciscinae (Teleostei, Cyprinidae) inferred from both mitochondrial and nuclear data. BMC Evol. Biol..

[15] Saito T (2018). Phylogeography of freshwater planorbid snails reveals diversification patterns in Eurasian continental islands. BMC Evol. Biol..

[16] Utevsky S, Trontelj P (2016). Phylogeography of the southern medicinal leech, *Hirudo verbana*: a response to Živić et al. (2015). Aquat. Ecol..

[17] Grabowski M, Jażdżewski K, Konopacka A (2007). Alien crustacea in polish waters—Amphipoda. Aquat. Invas..

[18] Kontula T, Väinölä R (2001). Postglacial colonization of Northern Europe by distinct phylogeographic lineages of the bullhead, *Cottus gobio*. Mol. Ecol..

[19] Mateus CS, Almeida PR, Mesquita N, Quintella BR, Alves MJ (2016). European lampreys: new insights on postglacial colonization, gene flow and speciation. PLoS ONE.

[20] Jażdżewski, K. Range extensions of some gammaridean species in European inland waters caused by human activity. 10–16 (1980).

[21] Bij de Vaate A, Jażdżewski K, Ketelaars HAM, Gollasch S, Van der Velde G (2002). Geographical patterns in range extension of Ponto-Caspian macroinvertebrate species in Europe. Can. J. Fish. Aquat. Sci..

[22] Panov VE (2009). Assessing the risks of aquatic species invasions via European inland waterways: from concepts to environmental indicators. Integr. Environ. Assess. Manag..

[23] Väinölä R (2008). Global diversity of amphipods (Amphipoda; Crustacea) in freshwater. Hydrobiologia.

[24] Weiss M, Leese F (2016). Widely distributed and regionally isolated! Drivers of genetic structure in *Gammarus fossarum* in a human-impacted landscape. BMC Evol. Biol..

[25] Weigand AM, Michler-Kozma D, Kuemmerlen M, Jourdan J (2020). Substantial differences in genetic diversity and spatial structuring among (cryptic) amphipod species in a mountainous river basin. Freshw. Biol..

[26] Rachalewski M, Banha F, Grabowski M, Anastácio PM (2013). Ectozoochory as a possible vector enhancing the spread of an alien amphipod *Crangonyx pseudogracilis*. Hydrobiologia.

[27] Peck SB (1975). Amphipod dispersal in the fur of aquatic mammals. Can. F. Nat..

[28] Sainte-Marie B (1991). A review of the reproductive bionomics of aquatic gammaridean amphipods: variation of life history traits with latitude, depth, salinity and superfamily. Hydrobiologia.

[29] Rewicz T, Grabowski M, MacNeil C, Bącela-Spychalska K (2014). The profile of a ‘perfect’ invader—the case of killer shrimp, *Dikerogammarus villosus*. Aquat. Invas..

[30] Vader W, Tandberg AHS (2019). Gammarid amphipods (Crustacea) in Norway, with a key to the species. Fauna Nor..

[31] Macdonald KS, Yampolsky L, Duffy JE (2005). Molecular and morphological evolution of the amphipod radiation of Lake Baikal. Mol. Phylogenet. Evol..

[32] Grabowski M, Wysocka A, Mamos T (2017). Molecular species delimitation methods provide new insight into taxonomy of the endemic gammarid species flock from the ancient Lake Ohrid. Zool. J. Linn. Soc..

[33] Jabłońska A, Wrzesińska W, Zawal A, Pešić V, Grabowski M (2020). Long-term within-basin isolation patterns, different conservation units, and interspecific mitochondrial DNA introgression in an amphipod endemic to the ancient Lake Skadar system, Balkan Peninsula. Freshw. Biol..

[34] Copilaş-Ciocianu D, Petrusek A (2015). The southwestern Carpathians as an ancient centre of diversity of freshwater gammarid amphipods: insights from the *Gammarus fossarum* species complex. Mol. Ecol..

[35] Copilaş-Ciocianu D, Petrusek A (2017). Phylogeography of a freshwater crustacean species complex reflects a long-gone archipelago. J. Biogeogr..

[36] Leuven RSEW (2009). The river Rhine: a global highway for dispersal of aquatic invasive species. Biol. Invas..

[37] Kelly DW, Muirhead JR, Heath DD, Macisaac HJ (2006). Contrasting patterns in genetic diversity following multiple invasions of fresh and brackish waters. Mol. Ecol..

[38] Panov V, Berezina N (2002). Invasive aquatic species of Europe. Distribution, impacts and management. Invas. Aquat. Species Eur. Distrib. Impacts Manag..

[39] Csabai Z (2020). Mass appearance of the Ponto-Caspian invader *Pontogammarus robustoides* in the River Tisza catchment: bypass in the southern invasion corridor?. Knowl. Manag. Aquat. Ecosyst..

[40] Rewicz T, Wattier R, Grabowski M, Rigaud T, Bącela-Spychalska K (2015). Out of the Black sea: phylogeography of the invasive killer shrimp *Dikerogammarus villosus* across Europe. PLoS ONE.

[41] Rewicz T (2017). The killer shrimp, *Dikerogammarus villosus*, invading European Alpine Lakes: a single main source but independent founder events with an overall loss of genetic diversity. Freshw. Biol..

[42] Jażdżewska AM (2020). Cryptic diversity and mtDNA phylogeography of the invasive demon shrimp, *Dikerogammarus haemobaphes* (Eichwald, 1841), in Europe. NeoBiota.

[43] Jażdżewski, K. & Roux, A. L. Biogéographie de Gammarus roeseli Gervais en Europe, en particulier répartition en France et en Pologne (1988).

[44] Piscart, C. & Bollache, L. *Crustacés amphipodes de surface : gammares d’eau douce.. Association Française de Limnologie, Introduction pratique à la systématique des organismes des eaux continentales de France* (2012).

[45] Paganelli D, Gazzola A, Marchini A, Sconfietti R (2015). The increasing distribution of *Gammarus roeselii* Gervais, 1835: first record of the non-indigenous freshwater amphipod in the sub-lacustrine Ticino River basin (Lombardy, Italy). Bioinvas. Rec..

[46] Karaman GS, Pinkster S (1977). Freshwater gammarus species from Europe, North Africa and adjacent regions of Asia (Crustacea-Amphipoda) Part II. *Gammarus roeseli*-group and related species. Bijdragen tot de dierkunde.

[47] Moret Y, Bollache L, Wattier R, Rigaud T (2007). Is the host or the parasite the most locally adapted in an amphipod-acanthocephalan relationship? A case study in a biological invasion context. Int. J. Parasitol..

[48] Copilaş-Ciocianu D, Borza P, Petrusek A (2020). Extensive variation in the morphological anti-predator defense mechanism of *Gammarus roeselii* Gervais, 1835 (Crustacea:Amphipoda). Freshw. Sci..

[49] Miller BJ, von der Heyden S, Gibbons MJ (2012). Significant population genetic structuring of the holoplanktic scyphozoan *Pelagia noctiluca* in the Atlantic Ocean. Afr. J. Mar. Sci..

[50] Brown WM, George M, Wilson AC (1979). Rapid evolution of animal mitochondrial DNA. Genetics.

[51] Kázmér M (1990). Birth, life and death of the Pannonian Lake. Palaeogeogr. Palaeoclimatol. Palaeoecol..

[52] Hewitt GM (1999). Post-glacial re-colonization of European biota. Biol. J. Linn. Soc..

[53] Rudolph K, Coleman CO, Mamos T, Grabowski M (2018). Description and post-glacial demography of *Gammarus jazdzewskii* sp. nov. (Crustacea: Amphipoda) from Central Europe. Syst. Biodivers..

[54] Copilaş-Ciocianu D, Fišer C, Borza P, Petrusek A (2018). Is subterranean lifestyle reversible? Independent and recent large-scale dispersal into surface waters by two species of the groundwater amphipod genus Niphargus. Mol. Phylogenet. Evol..

[55] Antal L (2016). Phylogenetic evidence for a new species of Barbus in the Danube River basin. Mol. Phylogenet. Evol..

[56] Walker MJC (1995). Climatic changes in Europe during the last glacial/interglacial transition. Quat. Int..

[57] Pawłowski D (2016). The response of flood-plain ecosystems to the Late Glacial and Early Holocene hydrological changes: a case study from a small Central European river valley. CATENA.

[58] Notebaert B, Verstraeten G (2010). Sensitivity of West and Central European river systems to environmental changes during the Holocene: a review. Earth Sci. Rev..

[59] Gibling MR (2018). River systems and the anthropocene: a late pleistocene and holocene timeline for human influence. Quaternary.

[60] Gherardi, F. *Biological invaders in inland waters: profiles, distribution, and threats.*10.1007/978-1-4020-6029-8 (2007).

[61] Jazdzewski K, Konopacka A, Grabowski M (2004). Recent drastic changes in the gammarid fauna (Crustacea, Amphipoda) of the Vistula River deltaic system in Poland caused by alien invaders. Divers. Distrib..

[62] Jourdan J, Piro K, Weigand A, Plath M (2019). Small-scale phenotypic differentiation along complex stream gradients in a non-native amphipod. Front. Zool..

[63] Mauchart P, Bereczki C, Ortmann-Ajkai A, Csabai Z, Szivák I (2014). Niche segregation between two closely related Gammarids (Crustacea, Amphipoda)—native vs. naturalised non-native species. Crustaceana.

[64] Lagrue C (2011). Interspecific differences in drift behaviour between the native *Gammarus pulex* and the exotic *Gammarus roeseli* and possible implications for the invader’s success. Biol. Invas..

[65] Pöckl M, Humpesch UH (1990). Intra- and inter-specific variations in egg survival and brood development time for Austrian populations of *Gammarus fossarum* and *G. roeseli* (Crustacea: Amphipoda). Freshw. Biol..

[66] Pöckl, M. Effects of temperature, age and body size on moulting and growth in the freshwater amphipods *Gammarus fossarum* and *G. roeseli*. 10.1111/j.1365-2427.1992.tb00534.x (1992).

[67] Pöckl M (1993). Reproductive potential and lifetime potential fecundity of the freshwater amphipods *Gammarus fossarum* and *G. roeseli* in Austrian streams and rivers. Freshw. Biol..

[68] Pöckl M, Webb BW, Sutcliffe DW (2003). Life history and reproductive capacity of *Gammarus fossarum* and *G. roeseli* (Crustacea: Amphipoda) under naturally fluctuating water temperatures: a simulation study. Freshw. Biol..

[69] Aguilera-Muñoz F, Lafarga-Cruz F, Gallardo-Escárate C (2009). Molecular analysis in Chilean commercial gastropods based on 16S rRNA, COI and ITS1-5.8S rDNA-ITS2 sequences. Gayana (Concepción).

[70] Alvarez JM, Hoy MA (2002). Evaluation of the ribosomal ITS2 DNA sequences in separating closely related populations of the *Parasitoid Ageniaspis* (Hymenoptera: Encyrtidae) article. Ann. Entomol. Soc. Am..

[71] Wesson DM, McLain DK, Oliver JH, Piesman J, Collins FH (1993). Investigation of the validity of species status of *Ixodes dammini* (Acari: Ixodidae) using rDNA. Proc. Natl. Acad. Sci. U. S. A..

[72] Tang J, Toè L, Back C, Unnasch TR (1996). Intra-specific heterogeneity of the rDNA internal transcribed spacer in the *Simulium damnosum* (Diptera: Simuliidae) complex. Mol. Biol. Evol..

[73] Palandačić A, Bravničar J, Zupančić P, Šanda R, Snoj A (2015). Molecular data suggest a multispecies complex of Phoxinus (Cyprinidae) in the Western Balkan Peninsula. Mol. Phylogenet. Evol..

[74] Vucić M, Jelić D, Žutinić P, Grandjean F, Jelić M (2018). Distribution of Eurasian minnows (Phoxinus : Cypriniformes) in the Western Balkans. Knowl. Manag. Aquat. Ecosyst..

[75] Buj I (2020). Peculiar occurrence of *Cobitis bilineata* Canestrini, 1865 and *Sabanejewia larvata* (De Filippi, 1859) (Cobitidae, Actinopteri) in the Danube River basin in Croatia. Fundam. Appl. Limnol..

[76] Manning JT (1975). Male discrimination and investment in *Asellus aquaticus* (L.) and *A. meridianus* Racovitsza (Crustacea: Isopoda). Behaviour.

[77] Bollache L, Cézilly F (2004). Sexual selection on male body size and assortative pairing in *Gammarus pulex* (Crustacea: Amphipoda): field surveys and laboratory experiments. J. Zool..

[78] Cornet S, Luquet G, Bollache L (2012). Influence of female moulting status on pairing decisions and size-assortative mating in amphipods. J. Zool..

[79] Grabner DS (2015). Invaders, natives and their enemies: distribution patterns of amphipods and their microsporidian parasites in the Ruhr Metropolis, Germany. Parasites Vectors.

[80] Karaman, G. S. & Pinkster, S. Freshwater gammarus species from Europe, North Africa and adjacent regions of Asia (Crustacea-Amphipoda) Part I. *Gammarus pulex*—group and related species (1977).

[81] Karaman, G. S. & Pinkster, S. Freshwater gammarus species from Europe, North Africa and adjacent regions of Asia (Crustacea-Amphipoda). Part III. *Gammarus balcanicus*—group and related species (1987).

[82] Hillis DM, Moritz C (1996). Molecular Systematics.

[83] Altschul SF, Gish W, Miller W, Myers EW, Lipman DJ (1990). Basic local alignment search tool. J. Mol. Biol..

[84] Kearse M (2012). Geneious basic: an integrated and extendable desktop software platform for the organization and analysis of sequence data. Bioinformatics.

[85] Sequencher version 5.4.6 DNA sequence analysis software, Gene Codes Corporation, Ann Arbor, MI USA https://www.genecodes.com.

[86] Rozas J (2017). DnaSP 6: DNA sequence polymorphism analysis of large data sets. Mol. Biol. Evol..

[87] Ratnasingham S, Hebert PDN (2007). The barcode of life data system. Mol. Ecol. Notes.

[88] Bouckaert R (2014). BEAST 2: a software platform for bayesian evolutionary analysis. PLoS Comput. Biol..

[89] Bouckaert RR, Drummond AJ (2017). bModelTest: Bayesian phylogenetic site model averaging and model comparison. BMC Evol. Biol..

[90] Rambaut, A., Suchard, M. A., Xie, D. & Drummond, A. J. Tracer v1.6. Available at https://beast.bio.ed.ac.uk/Tracer (2014).

[91] Kumar S, Stecher G, Tamura K (2016). MEGA7: molecular evolutionary genetics analysis version 7.0 for bigger datasets. Mol. Biol. Evol..

[92] Leigh JW, Bryant D (2015). POPART: full-feature software for haplotype network construction. Methods Ecol. Evol..

[93] Tajima, F. Statistical Method for Testing the Neutral Mutation Hypothesis by DNA Polymorphism. (1989). 10.1093/genetics/123.3.585 PMC1203831 2513255

[94] Fu YX (1996). New statistical tests of neutrality for DNA samples from a population. Genetics.

[95] Excoffier L, Lischer HEL (2010). Arlequin suite ver 3.5: a new series of programs to perform population genetics analyses under Linux and Windows. Mol. Ecol. Resour..

[96] Copilaş-Ciocianu D, Grabowski M, Parvulescu L, Petrusek A (2014). Zoogeography of epigean freshwater Amphipoda (Crustacea) in Romania: fragmented distributions and wide altitudinal variability. Zootaxa.

