# Supplementary material for: Successful post-glacial colonization of Europe by single lineage of freshwater amphipod from its Pannonian Plio-Pleistocene diversification hotspot
Source: Sci Rep. 2020 Oct 29;10:18695. doi: 10.1038/s41598-020-75568-7 (PMC7596225; doi:10.1038/s41598-020-75568-7)
Supplement: Supplementary file 1 — Supplementary Information [file 41598_2020_75568_MOESM1_ESM.docx]

**Supplementary Material**

**Successful post-glacial colonization of Europe by single lineage of freshwater amphipod from its Pannonian Plio-Pleistocene diversification hotspot**

**Hedvig Csapó**^1,2,3^**, Paula Krzywoźniak**^2^**, Michał Grabowski**^2^**, Remi Wattier**^4^**, Karolina Bącela-Spychalska**^2^**, Tomasz Mamos**^2,5^**, Mišel Jelić**^6^**, Tomasz Rewicz**^2,7^*****

^1^Institute of Oceanology Polish Academy of Sciences, Sopot, 81-712, Poland

^2^University of Lodz, Faculty of Biology & Environmental Protection, Department of Invertebrate Zoology and Hydrobiology, Łódź, 90-237, Poland

^3^University of Pecs, Faculty of Sciences, Department of Hydrobiology, Pecs, 7634, Hungary

^4^UMR CNRS 6282 Biogéosciences, Université Bourgogne Franche Comté, Dijon, 21000, France

^5^University of Basel, Zoological Institute, Basel, 4051, Switzerland

^6^Varaždin City Museum, Department of Natural Sciences, Varaždin, 42000, Croatia

^7^University of Guelph, Centre for Biodiversity Genomics, Guelph, Ontario, N1G 2W1, Canada

**Corresponding author:** tomasz.rewicz@biol.uni.lodz.pl

Supplementary Table S1. Primer pair used for PCR amplification

|  | **Forward** | | **Reverse** | |  |
| --- | --- | --- | --- | --- | --- |
| **Marker** | **name** | **sequence** | **name** | **sequence** | **Reference** |
| **COI** | **LCO1490** | GGTCAACAAATCATAAAGATATTGG | **HCO2198** | TAAACTTCAGGGTGACCAAAAAATCA | **Folmer et al. (1994)]** |
|  | **LCO1490-JJ** | CHACWAAYCATAAAGATATYGG | **HCO2198-JJ** | AWACTTCVGGRTGVCCAAARAATCA | **Astrin & Stüben (2008)** |
|  | **UCOIF** | TAWACTTCDGGRTGRCCRAAAAAYCA | **UCOIR** | ACWAAYCAYAAAGAYATYGG | **Costa et al. (2009)** |
|  | **COIGrF** | GCTAGHGCCGTAGGYACATC | **COIGrR1** | AAATARATGYTGRTAAAGAATAGG | **This study** |
|  |  |  | **COIGrR2** | RAATARGTGYTGGTACAGAATAGG | **Grabowski et al. (2017)** |
| **ITS2** |  | ACTCTGAGCGGTGGATCACT |  | TCCAAGCTCCATTGGCTTAT | **Flot et al. (2003)** |

Folmer, O., Black, M., Hoeh, W., Lutz, R. & Vrijenhoek, R. DNA primers for amplification of mitochondrial cytochrome c oxidase subunit I from diverse metazoan invertebrates. Mol. Mar. Biol. Biotechnol. 3, 294–299. https://doi.org/10.1071/ZO9660275 (1994).

Astrin, J. J. & Stüben, P. E. Phylogeny in cryptic weevils: molecules, morphology and new genera of western Palaearctic Cryptorhynchinae (Coleoptera: Curculionidae). Invertebr. Syst. 22, 503–522. https://doi.org/10.1071/IS07057 (2008).

Costa, F. O., Henzler, C. M., Lunt, D. H., Whiteley, N. M. & Rock, J. Probing marine Gammarus (Amphipoda) taxonomy with DNA barcodes. Syst. Biodivers. 7, 365–379. https://doi.org/10.1017/S1477200009990120 (2009).

Grabowski M, Mamos T, Bącela-Spychalska K, Rewicz T, & Wattier RA Neogene paleogeography provides context for understanding the origin and spatial distribution of cryptic diversity in a widespread Balkan freshwater amphipod. PeerJ 5:e3016 (2017).

Flot, J. F., Wörheide, G. & Dattagupta, S. Unsuspected diversity of Niphargus amphipods in the chemoautotrophic cave ecosystem of Frasassi, central Italy. BMC Evol. Biol. https://doi.org/10.1186/1471-2148-10-171 (2010).


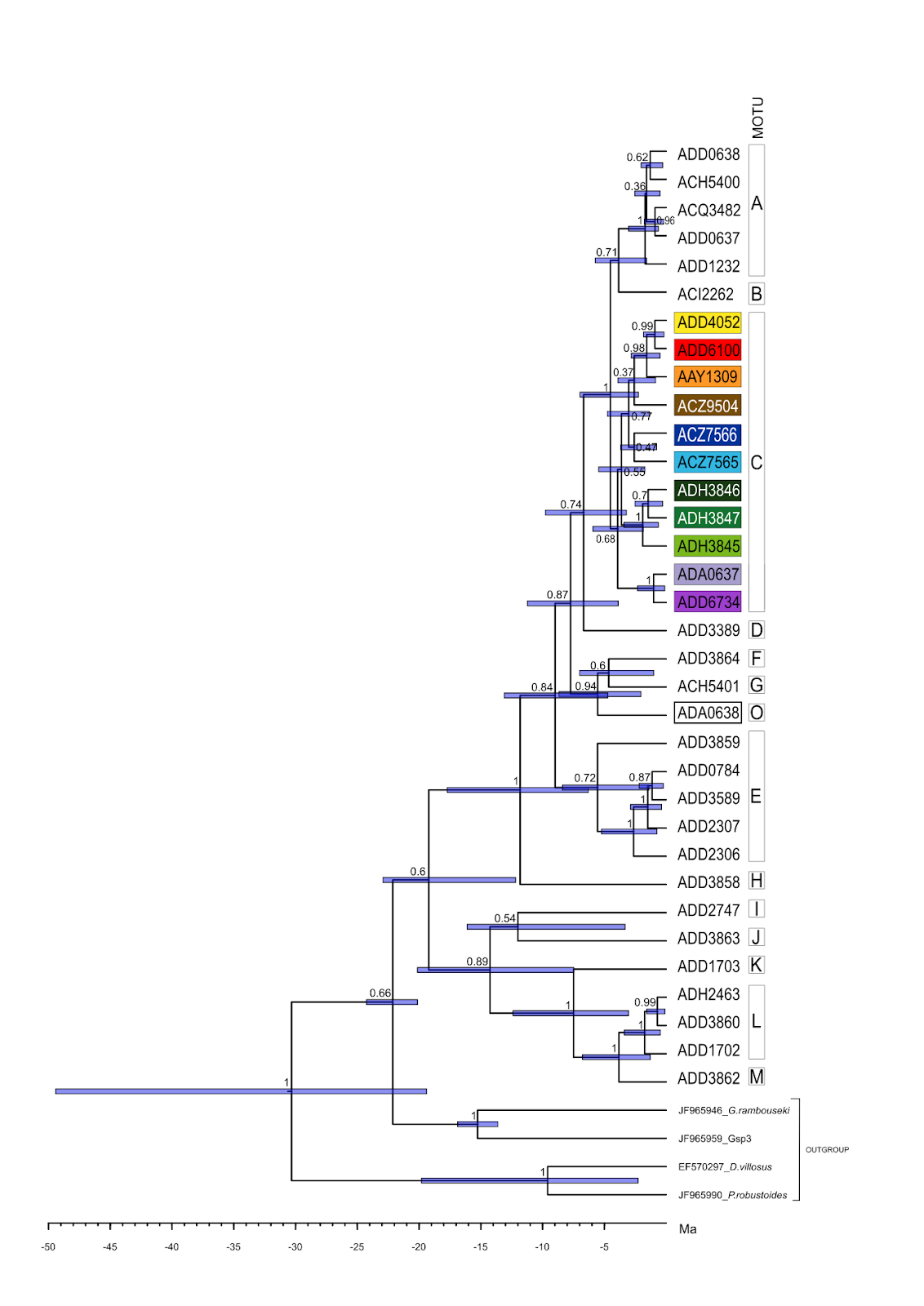


Supplementary Figure S1: Bayesian tree reconstructed from representative sequences of all COI BINs. Values at nodes indicate Posterior Probabilities (PP). Blue bars indicate credibility intervals of clade age.


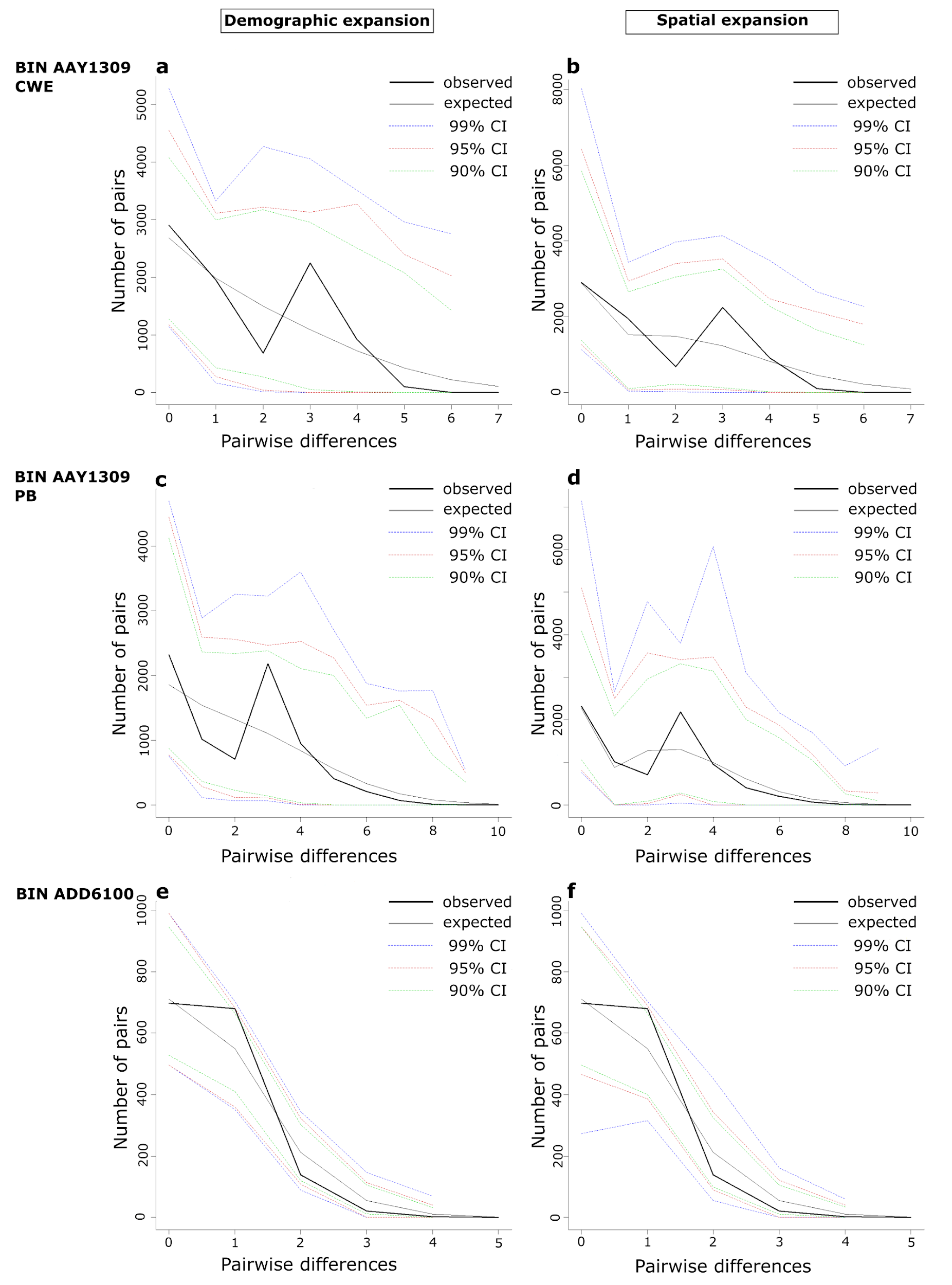


Supplementary Figure S2: Mismatch distribution graphs of the two most widely distributed COI BINs the study area. Graphs indicating the results of demographic and spatial expansion analyses of (A and B) BIN AAY1309 in CWE (C and D) BIN AAY1309 in PB and (E and F) ADD6100. The graphs show the expected and the observed distribution of pairwise differences between the sequences within the analyzed BINs. 90, 95 and 99% confidence intervals (CI) are indicated. CWE = Central Western Europe; PB = Pannonian Basin. Mismatch was generated using Arlequin 3.5.1.3 (http://cmpg.unibe.ch/software/arlequin35/) and visualized using R software (http://www.r-project.org).


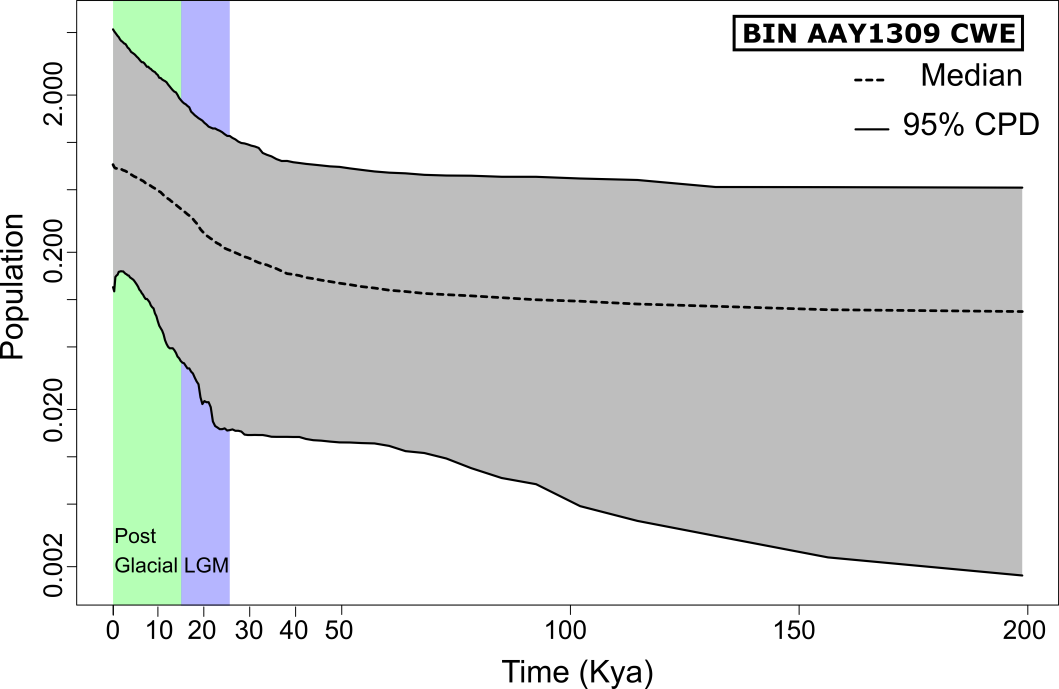


Supplementary Figure S3 Extended Bayesian Skyline Plot of BIN AAY1309 in CWE based on the combined analysis of COI+ITS2 markers. The plot shows the relative variation of effective population size time in Ky. Elevation of the graph indicates population expansion. CWE = Central Western Europe. EBSP were generated using BEAST 2.4.8 (https://www.beast2.org/) and visualized using R software (http://www.r-project.org).
